# Design of the Lifestyle Interventions for severe mentally ill Outpatients in the Netherlands (LION) trial; a cluster randomised controlled study of a multidimensional web tool intervention to improve cardiometabolic health in patients with severe mental illness

**DOI:** 10.1186/s12888-017-1265-7

**Published:** 2017-03-21

**Authors:** Anne Looijmans, Frederike Jörg, Richard Bruggeman, Robert Schoevers, Eva Corpeleijn

**Affiliations:** 1 Department of Epidemiology, University of Groningen, University Medical Center Groningen, Hanzeplein 1, PO box 30.001, 9700 RB Groningen, The Netherlands; 2 Rob Giel Research Centre, University of Groningen, University Medical Center Groningen, Groningen, The Netherlands; 3 Research Department, Friesland Mental Health Services, Leeuwarden, The Netherlands; 4 Department of Psychiatry, University of Groningen, University Medical Center Groningen, Groningen, The Netherlands

**Keywords:** Severe mental illness, Cardiometabolic health, Physical activity, Diet, Intervention, e-health, Web tool, Community-dwelling patients, Outpatients

## Abstract

**Background:**

The cardiometabolic health of persons with a severe mental illness (SMI) is alarming with obesity rates of 45-55% and diabetes type 2 rates of 10-15%. Unhealthy lifestyle behaviours play a large role in this. Despite the multidisciplinary guideline for SMI patients recommending to monitor and address patients’ lifestyle, most mental health care professionals have limited lifestyle-related knowledge and skills, and (lifestyle) treatment protocols are lacking. Evidence-based practical lifestyle tools may support both patients and staff in improving patients’ lifestyle. This paper describes the Lifestyle Interventions for severe mentally ill Outpatients in the Netherlands (LION) trial, to investigate whether a multidimensional lifestyle intervention using a web tool can be effective in improving cardiometabolic health in SMI patients.

**Methods/Design:**

The LION study is a 12-month pragmatic single-blind multi-site cluster randomised controlled trial. 21 Flexible Assertive Community Treatment (ACT) teams and eight sheltered living teams of five mental health organizations in the Netherlands are invited to participate. Per team, nurses are trained in motivational interviewing and use of the multidimensional web tool, covering lifestyle behaviour awareness, lifestyle knowledge, motivation and goal setting. Nurses coach patients to change their lifestyle using the web tool, motivational interviewing and stages-of-change techniques during biweekly sessions in a) assessing current lifestyle behaviour using the traffic light method (healthy behaviours colour green, unhealthy behaviours colour red), b) creating a lifestyle plan with maximum three attainable lifestyle goals and c) discussing the lifestyle plan regularly. The study population is SMI patients and statistical inference is on patient level using multilevel analyses. Primary outcome is waist circumference and other cardiometabolic risk factors after six and twelve months intervention, which are measured as part of routine outcome monitoring using standard protocols. Secondary outcomes include depressive and negative symptoms, cost-effectiveness, and barriers and facilitators in intervention implementation.

**Discussion:**

Adequate health care should target both mental health and lifestyle behaviours in SMI patients. This trial contributes by studying a 12-month multidimensional lifestyle intervention as a potential evidence based (nursing) tool for targeting multiple lifestyle behaviours in SMI patients.

**Trial registration:**

Nederlands Trialregister NTR3765 (trialregister.nl; registered 21 December 2012).

## Background

The cardiometabolic health of persons with a severe mental illness (SMI), such as schizophrenia, other psychotic or bipolar disorders, is alarming with obesity rates of 45-55% and type 2 diabetes rates of 10-15% [1]. This is up to four times higher than in the general population of comparable age [1]. The increased risk in SMI patients is associated with their illness (negative and depressive symptoms lead to disinterests in and lower levels of autonomous motivation towards physical activity [2, 3]), their treatment (antipsychotic medication, inadequate somatic treatment) and lifestyle factors (e.g. lack of exercise, unhealthy diet, smoking) [1, 4].

In mental as well as in general health care, SMI patients may receive insufficient attention for their physical condition [5]. In the Netherlands, screening of somatic and mental health on a regular base is now obligatory for SMI patients according to the multidisciplinary guideline [6]. However, somatic screening results indicating increased risk of negative health outcomes are seldom translated into (adequate) treatment [7]. General practitioners working with these patients may lack knowledge of this specific population. On the other side, psychiatrists and other mental health professionals may lack knowledge and expertise in addressing lifestyle issues. Due to their knowledge on the SMI population and the frequent contacts, mental health nurses (MH nurse) are assumed to be the most adequate persons to address lifestyle behaviour change in SMI patients. Therefore, evidence-based lifestyle tools that provide MH nurses with knowledge, techniques and practical skills to stimulate patients in behaviour change are needed.

Lifestyle interventions have been shown to be effective in the reduction of body weight [8] and cardiometabolic risk factors such as waist circumference, triglycerides and fasting glucose in adults with SMI [9, 10]. However, the quality of studies on the effectiveness of lifestyle interventions in the SMI population is rather low, samples are small and results are inconsistent [10, 11], although one well-designed relatively large intervention RCT has recently been published [12]. Systematic reviews on lifestyle interventions in different populations indicate that, to be effective, a lifestyle intervention should contain at least three key components: exercise, diet and behavioural therapy [11]. Behavioural therapy strategies that enhance individual behavioural change include improving self-management skills such as tailoring information to the individual, identifying (lifestyle) areas for improvement, goal setting, making action plans, giving personalized feedback to reinforce new behaviours and using social and environmental strategies to support change [13, 14]. However, most of these techniques have a limited effect, and only work well for patients who are motivated [15]. An approach to deal with unmotivated patients or patients who are not ready to change yet, is the motivational interviewing (MI) approach of Miller and Rollnick [16] combined with the stages-of-change from the transtheoretical model of Prochaska and DiClemente [17]. MI is a patient-centred counselling approach that targets behaviour change by addressing intrinsic motivation. MI seems more effective than traditional methods in targeting lifestyle change [18]. It has been shown to be effective in improving weight status, Body Mass Index (BMI) and cholesterol levels of overweight and obese adults and of clients in a broad range of other domains [19, 20]. According to the stages-of-change from the transtheoretical model, patients’ level of motivation and self-efficacy to change is reflected in one of the five stages of change [21, 22]: the precontemplation, contemplation, preparation, action or maintenance stage, ranging from no intention to change till the motivation to maintain behaviour change. Treatment (or intervention) should adapt to a patient’s stage-of-change in order to increase intrinsic motivation for behaviour change [21]. A combination of action planning with feedback and a motivational stages-of-change approach is believed to be effective in behavioural change in SMI patients [23]. In addition, mental care is nowadays more rooted in the community and therefore more depending on SMI patients’ peers, families and environment. Therefore, peer and family support is considered an essential component for successful intervention implementation.

In the Lifestyle Interventions for severe mentally ill Outpatients in the Netherlands (LION) trial, we propose a patient-centred multidimensional intervention using a web tool consisting of several of the above described successful intervention components, e.g. raising awareness of own lifestyle behaviours, goal setting, addressing motivation to change, personalized feedback, integrating support of friends and family and searching for healthy lifestyle activities in local communities (e.g. local sport clubs). An advantage of the intervention is that the tool addresses patients’ level of motivation (stage-of-change) to change diet and physical activity levels and that nurses are trained in motivational interviewing. This combination makes the intervention suitable for patients who do not seem motivated to change their lifestyle, indicating the intervention is considered eligible for more or less every patient. Due to the feasible character of the intervention, this trial will aim for a large sample size (~N = 250). Another unique feature of this trial is the pragmatic character of the intervention. Often, lifestyle interventions are implemented by external staff in strictly controlled conditions, recruiting the most motivated patients [8, 24]. In regular care however, staff with different levels of expertise will need to implement the intervention with available resources (e.g. time, budget), a high workload with competing priorities, and working with patients who may be unmotivated [8, 13]. This trial will show outcomes with high external validity of a lifestyle intervention implemented in a real-world care setting.

The pilot study seems promising: after three months intervention, patients receiving the multidimensional lifestyle intervention (*N* = 20) lost on average three kilograms of body weight compared to care-as-usual (CAU) controls, performed more physical activity and rated their general well-being as better than patients receiving CAU (*N* = 20) [25]. Patients mentioned as enabling factors the role of nurses in stimulating a healthy lifestyle, and that more physical activity made them feel better, which enabled them to change other lifestyle factors as well. The intervention was well appreciated by patients and staff.

### Aims of the trial

The aims of current pragmatic trial are to test whether a 12-month multidimensional lifestyle intervention, including aspects of increased awareness of own lifestyle and related risks, motivation, self-management, diet, exercise, and a supportive environment, is (cost-)effective in reducing cardiometabolic health and decreases depressive and negative symptoms. Also, barriers and facilitators in implementing the intervention on nurse and patient level will be explored.

The primary research question is:

1. Is a 12-month multi-dimensional lifestyle approach including a web tool for SMI patients effective in improving or stabilising abdominal obesity (waist circumference) and other cardiometabolic risk factors in SMI patients after six and twelve months intervention compared to care as usual?

Secondary research questions are:

2. Is a 12-month multi-dimensional lifestyle approach including a web tool for SMI patients effective in reducing depressive and negative symptoms in SMI patients after six and twelve months intervention compared to care as usual?
3. Is a 12-month multi-dimensional lifestyle approach including a web tool for SMI patients aimed at improving or stabilising abdominal obesity (waist circumference) and other cardiometabolic risk factors in SMI patients after six and twelve months intervention compared to care as usual, cost-effective?
4. What barriers and facilitators on nurse and patient level affect implementation of the 12-month multi-dimensional lifestyle approach?

We hypothesize that this 12-months multi-dimensional lifestyle approach will improve cardiometabolic risk factors compared to patients who receive care as usual. Specifically, we expect the intervention to reduce waist circumference (WC), Body Mass Index (BMI) and Metabolic Syndrome Z-score (MS Z-score) after six and twelve months intervention because we expect that patients will try to increase their physical activity levels and improve their dietary habits. We expect that, through the intervention, patients will increase levels of physical activity and experience improvements in self-management skills and thereby improving self-efficacy [3, 14, 26], leading to a decrease in depressive [27] and negative symptoms [28] (i.e. lower depressive and negative symptoms scores). We hypothesize that the intervention will be cost-effective as costs will be relatively low (training of staff) while the physical and mental health of SMI patients will improve. Improvements in health, due to the increased self-management and increased exercise, might lead to less psychotropic drug use, such as antidepressants and anxiolytics [27, 28]. Finally, we will explore what barriers and facilitators on patient and nurse level have an influence on intervention implementation.

## Methods

The Lifestyle Interventions for severe mentally ill Outpatients in the Netherlands (LION) trial is a pragmatic single-blind multi-site cluster randomised controlled trial (RCT). Details are described below according to the SPIRIT 2013 statement [29]. The study was approved by the Medical Ethical Committee of the University Medical Center Groningen. The trial is registered in the Dutch Trial Registry NTR3765 (www.trialregister.nl, 21 December 2012).

### Study setting

**Fig. 1:**
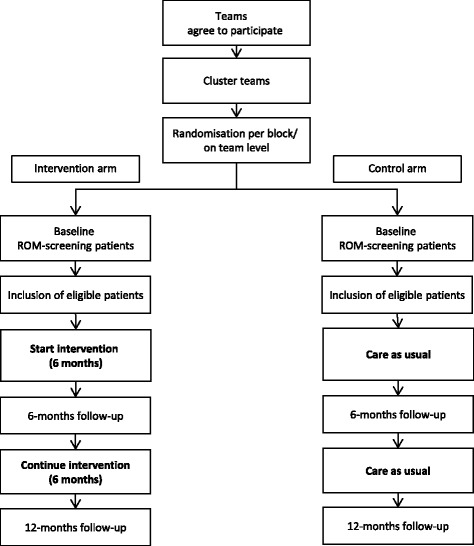
Flowchart of the LION-study

Mental health care for severe mentally ill (SMI) outpatients is organized by a Flexible Assertive Community Treatment (FACT) team with MH nurses. FACT means that patient care is outreaching, takes place in the community (patients’ own environment) and ranges from high intensive (24 h) treatment to low intension support – for a detailed description of FACT, see [30, 31]. Patients living in sheltered facilities receive a combination of housing and services in the community. 21 FACT and eight sheltered facility teams serving SMI patients of five mental health care organizations in the North of the Netherlands, an area covering 2.8 million inhabitants, are invited for this study. Per team, nurses are invited to participate. All teams per organization are matched based on caseload size, mean age of patients, mean duration of admission of patients, most frequent diagnosis and location (urban or rural). After matching, teams are randomly allocated to the control or intervention arm by means of a random number generator by a researcher of the research team not involved in training of staff and recruitment of patients (see Fig. 1 for flowchart of the study). To minimise spill-over, randomisation is on team level, although inclusion of study participants and statistical inference are on patient level.

### Participants

The study population consists of community-dwelling SMI patients and SMI patients living in sheltered facilities. Of this population, approximately 75% of the patients is diagnosed with a psychotic disorder, 15% with bipolar disorder and approximately 10% with complex personality disorders. In the North of the Netherlands, SMI patients are invited for annual Routine Outcome Monitoring screenings as part of standard care, consisting of a physical examination, a lab test and psychosocial measures. Patients are invited for the LION study when ROM screening outcomes indicate at least one of the following risk factors for metabolic syndrome: waist circumference > 88/102 cm (females/males); fasting glucose > 5.6 mmol/L or HbA1c > 5.7%; BMI > 25 kg/m^2^. Exclusion criteria are being pregnant, a BMI < 19 kg/m^2^, being primarily diagnosed with Korsakov syndrome or having a physical impairment which makes daily physical activity impossible. When patients are eligible for the study, they receive a detailed information letter from their case manager and, if willing to participate, sign informed consent.

The main objective of this trial is to detect an abdominal weight loss having public health significance. Previous work has indicated that 5-10 cm reduction in waist circumference (WC) is considered a realistic guideline with a high probability of health benefits [32]. For power calculations, we assumed 10% dropout rate. To include 250 participants, a 10% extra will be needed resulting in a total of 275 patients. Under these assumptions, and assuming an SD of 16.3 cm based on pilot data from this population, for two-sided 0.05-level tests of the null hypothesis, the study should provide approximately 80% power for detecting a difference of 5.8 cm in WC at 12 months between intervention and control groups. In addition, the study will have the same power to detect a reduction of 0.6 mmol/L in plasma glucose, given an SD of 1.7 mmol/L.

### Procedure

After patients have signed informed consent, the research coordinator creates a web tool account for the patient. Hereafter, patient and nurse start the intervention by using the web tool ‘Traffic Light Method for somatic screening and lifestyle’ (TLM). The web tool is used during regular care visits, which take place, on average, once every two weeks. During the first visit, patient and nurse map out lifestyle behaviour in the web tool; during later visits they update progress (follow-up) reports (details below). Filling in a follow-up report each biweekly care visit in the follow-up phase is estimated to take 15 min. Six months after start of the intervention, the six-months measures take place. Hereafter, patient and nurse start again with the lifestyle behaviour screening and creating a lifestyle plan, followed by the follow-up phase until the end of the trial (12-months measure).

### Intervention

The intervention in this trial is a 12-month multidimensional, patient-centred lifestyle intervention, including use of the web tool ‘Traffic Light Method for somatic screening and lifestyle’ (TLM), which supports behaviour change in various phases. The five most important features of the intervention are presented in Table 1.

**Table 1:** Five important features of the multidimensional lifestyle intervention using a web tool in the LION study

Feature	Description
1	Patients’ readiness for behaviour change is not a prerequisite for starting the intervention. Nurses encourage behaviour change by making use of the stages-of-change of the transtheoretical model [17] and motivational interviewing [16].
2	Patient-centeredness: patients decide if and what behaviour he/she wants to change, creates his/her own lifestyle plan with realistic goals and support. The tool can also be used by patients themselves to enhance self-management.
3	Because diet and physical activity are key components of a healthy lifestyle, these components are combined with behavioural change counselling; for an intervention to be effective, these three ingredients should be included [11].
4	Active support of the patient’s family and friends, incorporated in the lifestyle plan.
5	Nurses are trained to not only support patients in their behaviour change but also work behind the scenes to create a healthier environment: organise accessible exercise opportunities, raise team support for a healthier lifestyle in patients and share up to date lifestyle knowledge with the team, and raise awareness among other health care professionals (e.g. GP’s) of the increased cardiovascular risk of most SMI patients.

The 12-month intervention will be delivered by MH nurses. Before the start of the study, nurses will receive one day of training on (a) basic components of motivational interviewing [16] and the stage of change model [33], (b) side effects of psychotropic medication, (c) lifestyle of and risks for SMI patients, (d) working with TLM, and (e) environmental factors that affect effectively working with TLM (e.g. health behaviour of staff members themselves or the availability of unhealthy products in the home environment) – see Meijel (2015) [25] for more details. In addition, the study protocol will be explained. After three months, an evaluation session is planned to discuss obstacles with the tool, obstacles in motivating patients to participate and to recollect study protocol. Trained LION nurses are, due to the nature of the intervention, not blind for study allocation.

#### Traffic Light Method for somatic screening and lifestyle (TLM)

**Fig. 2:**
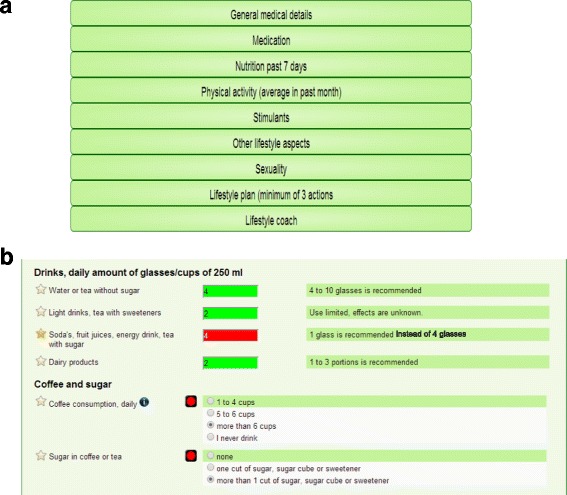
**a** and **b** Preview of the web tool Traffic Light Method (TLM). Legend: **a** the starting page of the lifestyle behaviour screening representing the domains discussed in the Traffic Light Method (TLM) web tool; **b** examples of questions in the dietary domain within the lifestyle behaviour screening with built-in features to increase awareness (colouring according to risk profile) and knowledge (green bars presenting healthy reference values according to (inter)national guidelines)

The Traffic Light Method (TLM) is a web tool originally developed as a practical tool for nurses and patients in one Dutch mental health care organization (GGz Centraal) and further advanced by a small spin-off company (Charly Green, Bilthoven, The Netherlands). It is based on the current state of the art of effective interventions and (inter)national guidelines on healthy lifestyle behaviour. During development, it was extensively reviewed by experts from the field in a Delphi panel and its use was optimized in a pilot study at GGz Centraal [25]. The web tool is, after registration, available online (www.leefstijlinbeeld.nl; for a preview, see Fig. 2a and b).

The TLM consists of two parts: (I) a lifestyle behaviour screening followed by creating a lifestyle plan and (II) a follow-up phase. In the lifestyle behaviour screening, the patient, together with a nurse, answers questions on several health and lifestyle related domains, see Table 2 for an overview of these domains. The Traffic Light Method displays a risk profile with all lifestyle behaviours in green, orange or red, depending on the level of risk. The patient creates, while being coached by the nurse, a lifestyle plan containing maximum three attainable lifestyle goals. During the subsequent follow-up phase, nurse and patient will systematically evaluate the patient’s progress in achieving the lifestyle goals described in the lifestyle plan. This will be done biweekly during regular care visits for approximately 15 min. In order to enhance and stimulate behaviour change, several techniques are built in the web tool. The aims of the lifestyle behaviour screening and the follow-up phase are presented in Table 3.

**Table 2:** Domains and subdomains in web tool Traffic Light Method (TLM)

Domain	Subdomains
(a) General medical information	1. Physical measures^a^
	2. Measures from lab test^a^
	3. Physical diseases and handicaps
	4. Rating own health
(b) Use of medication	1. Satisfaction with medication use
	2. Somatic medication
	3. Psychiatric medication
	4. Freely available medication
(c) Dietary habits (last 7 days)	1. Satisfaction with own dietary behaviour
	2. Rating own dietary behaviour
	3. Assessing stage-of-change for dietary behaviour change
	4. Assessing dietary habits
(d) Physical activity (last month)	1. Satisfaction with own physical activity
	2. Rating own physical activity
	3. Assessing physical activity with SQUASH questionnaire
	4. Assessing stage-of-change for physical activity behaviour change
	5. Sedentary behaviour
(e) Use of stimulants	1. Disadvantages of dependence on substances
	2. History of substance abuse
	3. Use of alcohol
	4. Smoking behaviour
(f) Other lifestyle factors	1. Personal hygiene
	2. Relaxation
	3. Sleep behaviour
	4. Computer behaviour
	5. Social environment
(g) Sexuality	1. Condom use
	2. Sexually transmitted diseases
(h) Lifestyle plan^b^	

^a^ Measures are taken from the Routine Outcome Monitoring screening conducted within two months prior to the web tool assessment. ^b^ Only available for participants in the intervention group

**Table 3:** Aims for the lifestyle behaviour screening and the follow-up phase in the lifestyle intervention

Aim	Description of aim per phase
Lifestyle behaviour screening phase	
1	Identify unhealthy lifestyle behaviours. The tool uses a traffic light principle for a clear visible presentation of possible health risks related to certain lifestyle behaviours, with green colours representing behaviours with low or no health related risk and red colours representing behaviours with high health related risks (see Fig. 2b).
2	Increase patient’s and nurse’s knowledge of healthy lifestyle behaviours. The tool provides direct feedback on what healthy behaviours are according to (inter)national guidelines and gives additional information to increase patient’s and nurse’s knowledge on healthy lifestyle behaviours (see Fig. 2b).
3	Create awareness. Patients are challenged to discuss identified risk factors and nurses support patients in deciding what lifestyle behaviours to change. Nurses use MI and stages-of-change techniques to assist patients in identifying their problems and overcoming ambivalence or resistance to behaviour change. It is supported by regularly classifying the patient’s current stage-of-change.
4	Create a lifestyle plan with concrete and reachable goals. Based on the lifestyle anamnesis and discussion with the nurse, patients set maximum three goals to achieve according to the criteria of S.M.A.R.T.-goals [58]. The nurse’s role is to support patients in setting realistic goals. Patients explore which interventions are available and seem attractive, and what is needed to reach goals. Active self-management of patients is encouraged, support of family and friends is explored and, when available and deemed necessary, incorporated in the plan.
Follow-up phase	
6	Evaluating lifestyle goals systematically on a regular basis. During every regular care visit, a new follow-up file is uploaded and filled in by patient and nurse. By doing this, continuity is ensured and this repetitive character will lead to more sustainable behaviour change.
7	Barriers and facilitators in achieving lifestyle goals are indicated. Patient and nurse discuss which factors are helpful in achieving goals and which factors limit achieving goals in order to increase the success of achieving the goals in the following period. Again, nurses use motivational interviewing techniques and the stages-of-change of the transtheoretical model.

All information entered in the web tool can be printed as a personal booklet for the participant to share the information and his/her lifestyle plan with friends and family or to use healthy lifestyle information in daily life (such as when doing groceries, preparing food).

#### Implementation strategy

To increase the degree of implementation of the intervention, an implementation strategy was defined consisting of several components: 1) establish support from organizational management, 2) involve team management, 3) train MH nurses in using the web tool, motivational interviewing and the stage of change model, 4) plan a meeting with MH nurses and the trainer three months after training, 5) plan regular visits of research team one every three months, 6) send out newsletters to keep teams and nurses informed and involved.

### Control group

Patients in the control group participate in ROM screenings and results are discussed with the patient as part of standard care. Because data on lifestyle behaviours will be gathered from the lifestyle anamnesis part in the web tool, patients in the control group fill in the questions in the anamnesis part of the tool, but in blanc version in the web tool or on paper version; they do not receive any feedback or information via colours or education rules. In addition, patients in the control condition do not set up a lifestyle plan and therefore have no biweekly follow-up sessions. Nurses in the control group are instructed to give care as usual. This implies medical problems are tackled immediately according to protocol, while lifestyle guidance is more or less provided when patients wish to (based on ROM screenings).

### Outcomes

Measurements are performed on patient and staff level. An overview of all measurements at baseline, six and twelve months is given in Table 4.

**Table 4:** LION trial measurement overview

		Baseline	6 months	12 months
Measurements on patient level				
Routine Outcome Monitoring				
General information	Birth year, gender, diagnoses, year of first psychosis	X		
	Medication use	X		X
Physical measures	Height	X	X	X
	Weight	X	X	X
	Waist circumference	X	X	X
	Blood pressure (systolic, diastolic, pulse)	X	X	X
Lab test	Lipids (Total cholesterol, LDL-cholesterol, HDL-cholesterol, triglycerides)	X	X	X
	Glucose metabolism (glucose, HbA1c)	X	X	X
Psychological measures^b^	CDSS	X		X
	PANSS	X		X
	HoNOS	X		X
	MANSA	X		X
Cost-effectiveness^a^	Dutch care consumption questionnaire	X	X	X
	SF6D	X	X	X
Web tool TLM				
Lifestyle habits	Daily physical activity (SQUASH)	X	X	X
	Food frequency questionnaire (adapted to patient population)	X	X	X
Additional measure by research assistant				
	Physical activity (pedometers) and body fatness^c^	X	X	X
Measurements on staff level				
General information	Birth year, gender, level of education, number of years working in psychiatry, function	X		
Staff questionnaire	Knowledge on diet and physical activity, attitude towards lifestyle changes in patients, self-efficacy in addressing lifestyle issues with patients	X		X
	Daily physical activity (SQUASH)	X		X
	Food frequency questionnaire	X		X

^a^ Measures are not part of standard ROM screening but added to ROM screening for the purpose of this study. ^b^ The conducted psychosocial measures within the ROM protocol could vary per team, not all teams conduct every psychosocial measure. ^c^ Only conducted by one of the five health care organisations (GGZ Friesland)

#### Measurements on patient level

Most measures on patient level are conducted during the ROM screening, which is part standard care and of the scientific ongoing PHAMOUS (Pharmacotherapy Outcome and Monitoring Survey) cohort [34]. In mental health care organisations in the North of the Netherlands, it is routine care that ROM trained nurses invite patients annually for a ROM screening including somatic and psychosocial measures. Data of these measurements are reported in patients’ record forms and discussed with the patient. These data are stored in a large database and anonymized data are available for scientific research. This method was approved by the Medical Ethical Committee of the University Medical Center Groningen. For the LION study, data of two regular ROM screenings will be used for baseline and 12-months measures. An additional, short version of the ROM screening is scheduled six months after start of the intervention and patients will receive a small fee (€5,00/£4,30) for participation. ROM nurses carrying out the assessments are blinded for study allocation.

General data on birth year, gender, diagnosis, duration of illness and use of medication are derived from patient record forms.

#### Cardiometabolic health

The physical measurements include waist circumference, height, weight, pulse and systolic and diastolic blood pressure. Patients visit a (hospital) laboratory that collects a blood sample, if possible in fasting state, for levels of lipids (total cholesterol, LDL-cholesterol, HDL-cholesterol and triglycerides [all in mmol/L]) and glucose metabolism (glucose [mmol/L], HbA1c [%]). Measurements are taken following standard ROM protocols. Waist circumference (in cm) is measured in duplicate using a flexible nonstretching tape halfway between the iliac crest and lowest rib in standing position at the end of an expiration. Body weight is measured by calibrated scales (Seca, model 813) in light clothing without shoes or jackets. Measurements for height (in cm) will be available from multiple measurements of ROM nurses. The highest height will be used unless patients wear shoes, then the highest height without shoes is used. Pulse and systolic and diastolic blood pressure are measured after 5 min’ rest in sitting position, using a blood pressure monitor (BOSO medicus control).

#### Mental health

During an interview, trained nurses administer positive and negative symptoms with the PANSS (Positive and Negative Syndrome Scale [35]) and depressive symptoms with the CDSS (Calgary Depression Scale for Schizophrenia [36]). Prior to the interview, patients fill in the MANSA, a self-report questionnaire about patients’ Quality of Life [37] and uncertainties can be discussed during the interview. The HoNOS (Health of the Nations Outcome Scale [38]) is an observation scale of psycho-social functioning and is scored by the case manager or team.

#### Lifestyle habits

LION trained nurses assess lifestyle habits using the lifestyle behaviour screening part in the web tool TLM. Items in the TLM physical activity and nutritional domain serve both a measurement purpose as well as an intervention purpose. Daily physical activity is assessed using the Dutch validated SQUASH questionnaire [39]. Nutritional habits are estimated using a semi-quantitative food frequency questionnaire (FFQ) with items based on a screening questionnaire for healthy eating habits of the Netherlands Nutrition Center according to the Dutch guidelines for a healthy diet [40] and adapted to this population. The questionnaire will be used to assess changes in dietary habits on food group level. It is not specifically validated in SMI patients and can and will not be used to derive quantitative estimates of total energy, macro- or micronutrient intake.

#### Motivation to change

The stages-of-change for physical activity behaviour change and for dietary behaviour change are assessed based on the five phases of the stage-of-change model [17]. These stages indicating whether a patient is in the precontemplation (not ready to change), contemplation (thinking about possible change), preparation (preparing to change), action (carrying out changed behaviour) or maintenance phase (maintaining changes behaviour).

#### Cost-effectiveness parameters

Care consumption is estimated with the Dutch care consumption questionnaire [41], which is adapted to the context of the current study. Use of medication is derived from patient record forms. Quality adjusted life years (QALYs) will be the primary outcome measure in the cost-effectiveness analysis. In order to estimate QALYs, utility scores will be derived from the SF12, using the SF6D algorithm [42, 43].

#### Physical fitness

All patients of one organisation (GGZ Friesland) are invited to wear a pedometer (Yamax SW200 [44]) for at least seven days, reporting the total steps per day in a diary. A trained research assistant measures patients’ body fat percentage in standing position [45] by bioelectrical impedance analysis (BIA) in triplicate using a single-frequency bioimpedance analyzer (Model BIA 101, AKERN Srl, Italy) [46, 47]. In order to calculate the body fat percentage using a formula, height and weight are measured in accordance with previously described methods.

#### Web tool evaluation

After the intervention, participants’ perception of and satisfaction with the web tool is assessed by a questionnaire.

### Measurements on staff level

Staff members receive an online questionnaire at baseline and after the intervention is finished to gather information on, among other things, own lifestyle behaviours, attitudes towards lifestyle and process evaluations as potential determinants influencing intervention implementation. Data on birth year, level of education and experience are only collected at baseline.

#### Knowledge on diet and physical activity

Staff members answer questions to rate their knowledge on physical activity and diet, based on national guidelines regarding physical activity [48] and diet [40].

Attitudes, self-efficacy and frequency of performing lifestyle related activities. The questionnaire also addresses staff members’ attitudes toward lifestyle coaching for patients, rate their self-efficacy in lifestyle coaching and rate how often they perform lifestyle related activities with/for patients and the difficulty they experience with these activities [49]. Questions on attitude and self-efficacy are based on the ACE-model which describes the relationship between a persons’ attitudes, social influences and self-efficacy, and their behaviour. Questions are adapted to fit the study design and patient group [50, 51]

#### Lifestyle habits

Staff members’ daily physical activity is assessed with the SQUASH questionnaire [39] and their diet is assessed using a semi-quantitative food frequency questionnaire (FFQ) with items based on a screening questionnaire for healthy eating habits of the Netherlands Nutrition Center according to the Dutch guidelines for a healthy diet [40].

#### Web tool evaluation

After the intervention, nurses fill in a questionnaire about how they perceived working with the web tool.

### Statistical analysis

Variables will be presented as mean ± standard deviation (SD), and if not normally distributed as median [25th-75th percentiles], or N (%) for frequencies. Missing data are handled differently based on amount and type of missingness. If missing data can be well predicted by regression methods, multiple imputation will be considered. Otherwise, interpolation or replacement by study mean or median will be preferred over complete case analyses.

The primary outcome is the change in waist circumference (in cm) over time (from baseline to six and from baseline to twelve months) comparing the intervention group to the control group. This will be analysed with multilevel linear mixed models using teams as cluster. Analysis will be based on intention-to-treat principle. In per-protocol analyses, the intervention effect on cardiometabolic risk factors will be studied as described above, comparing participants with different degrees of intervention adherence to controls. Secondary study outcomes are analysed according to the same principles and techniques as the primary outcome. A priori sensitivity analyses are foreseen for participants’ age, gender and type of housing. In additional analyses, we will test whether the 12-months lifestyle intervention changes the level of motivation (stage-of-change) for changing diet of physical activity levels. An alpha of 0.05 is considered statistically significant.

## Discussion

Given the disturbingly high levels of metabolic diseases in SMI patients, and the associated risks of premature death [1], it is of high importance to develop lifestyle interventions that can effectively be implemented in regular care. The current study investigates whether a multidimensional lifestyle intervention using a practical lifestyle tool for mental health nurses to improve their knowledge, skills and expertise regarding healthy lifestyle behaviours and behaviour change in severe mentally ill patients, influences cardiometabolic risk factors of SMI patients in their caseload. The Traffic Light Method (TLM) tool aims for patients to increase lifestyle behaviour awareness and knowledge, improve self-management (setting lifestyle goals, receiving systematically feedback) and to involve friends and family in achieving lifestyle goals. The primary outcome of the study is waist circumference, considered the best predictor of abdominal fatness and cardiovascular disease [52], and other cardiometabolic risk factors. These measures are strongly associated with a range of negative health outcomes, such as type 2 diabetes, stroke and cardiovascular disease [53, 54].

### Strengths of the intervention

The motivational interviewing approach is a major strength of this intervention as it enables inclusion of all patients, regardless of their motivation to change their lifestyle behaviours. Addressing the (lack of) motivation as part of the intervention has been proven successful in improving medication adherence in persons with schizophrenia [55], altering substance (ab)use [56] and weight loss in (overweight and obese) adults [19, 20]. Because the intervention is implemented during regular care visits by their own MH nurse, large numbers of patients can benefit from the intervention.

In the intervention, the patient is taking the lead in creating a lifestyle plan and determining his/her lifestyle goals. Therefore, every patient is able to direct the lifestyle intervention in such a way that it contributes to his/her specific recovery wishes. This fits well within the recovery approach in which patients take control over their own recovery process and decide themselves which (lifestyle) behaviours they wish or need to change in order to recover [57]. In the field of mental health, the recovery approach fits well because of the person-centeredness, focus on improving quality of life besides solely reducing impairments of the mental illness and the acknowledgment of multiple possible pathways to recovery.

Another advantage of using the TLM web tool is that it systematically addresses a broad range of lifestyle behaviours instead of solely focussing on diet or physical activity. This gives patients the option to choose which lifestyle behaviour they wish to change. In addition, health professionals have expressed that lifestyle interventions should at least include the following lifestyle topics: “(1) healthy eating; including buying healthy foods on a budget, cooking skills and recipes, (2) the risks of weight gain and how to monitor weight, (3) exercise; what is available, physically possible, affordable and accessible, (4) dental hygiene, (5) substance misuse and (6) physical health monitoring such as blood checks” (p. 402) [23]. All mentioned components are present in TLM, therefore it can be considered a complete and comprehensive lifestyle intervention tool.

A last strength of the intervention is that the web tool constitutes an objective source of information that draws the attention of both patient and MH nurse to unhealthy lifestyle behaviours by presenting a risk profile and showing related healthy options. The nurse coaches the patient in the behavioural changes he/she wishes to make using MI and the stages-of-change techniques. Therefore, the MH nurse will not impose unwanted lifestyle advices, which is a benefit for the professional relationship between nurse and patient.

### Strengths of the study design

The study design for this trial has several advantages. First, by using regular ROM screenings and structurally inviting all patients with screening outcomes indicating at least one cardiometabolic risk factor, patient selection bias is minimal. Second, the intervention is feasible for a large number of patients (e.g. also unmotivated patients are eligible, implementation during regular care visits), leading to a large and highly representative study sample to be included. Third, follow-up of less motivated patients is feasible because of the routine ROM screenings, which will be performed routinely in all patients. Fourth, data collection is based on existing Routine Outcome Monitoring screenings infrastructures, which has several advantages: ROM nurses are well trained, baseline and follow-up measures are conducted by the same nurse, and additional time and costs of patients, nurses and researchers are limited. The ROM data collection covers a wide scope of measures, i.e. several physical measures and multiple psychosocial measures and, for this trial, only had to be extended with cost-effectiveness measures. Finally, the pragmatic character of the study, in which MH nurses carry out the intervention in hectic real word health care settings, will result in realistic and achievable intervention outcomes, representing outcomes with high external validity [8, 13].

### Potential risks for bias

Some potential risks for bias might be expected. First, although patient selection bias should be minimal, it is still possible that it is difficult for nurses to include patients that are unmotivated to change their lifestyles in a lifestyle intervention study. Motivated patients are expected to be more easily included, leading towards patient selection bias based on the level of motivation. Second, because patients determine which lifestyle behaviours they wish to change, it is possible that they target other somatic health outcomes (e.g. dental health, sleeping patterns) than the primary study outcome waist circumferences and other cardiometabolic risk factors. In this case, stating that the intervention does not seem effective in reducing the primary outcome might be a biased conclusion when patients wished to change other health outcomes and might have been successful in addressing these changes. Third, although ROM is implemented on all sites, it is possible that not all sites have the capacity to conduct all measures, leading to missing values. In addition, ROM nurses cannot collect blood samples themselves but send patients an invitation to visit a (hospital) laboratory. Although ROM nurses convince and remind patients to do so, patients might be reluctant to go to the laboratory, also leading to possible missing values.

### Changes in study design between obtaining funding and preparation of the study

In the period between obtaining funding and preparing the study, unexpected large changes in the organisation of mental health care took place. Budgets were restrained and care delivery shifted from specialists towards general mental health care, leading to necessary adjustments in study design. The initial sample size was estimated based on 64 nurses all including 10 patients leading to a target sample of 640 patients. The Medical Ethics Committee advised us to plan an extra 20% inclusion to account for clustering of the data, yielding a target sample of 768 patients. However, due to increased workload, inclusion of twelve patients per nurse seemed unfeasible. To compensate, we planned to train more nurses so that less patients per nurse need to be included, and we furthermore broadened inclusion criteria so that patients in sheltered living facilities could be included as well. As we now had many clusters (teams and nurses) and relatively few patients per nurse, it was not necessary anymore to account for clustering of the data in calculating the sample size. The funding agency (ZonMw) and the Medical Ethics Committee have approved the adjusted study design and adjusted final target sample size of 275 patients.

The somatic health of patients in mental health care can no longer be ignored. Changing lifestyle behaviours is difficult, but combining several successful components (e.g. motivational interviewing, stage-of-change, objective monitoring, self-management, support of peers and family etc.) into one multidimensional intervention might enhance successful, sustainable lifestyle changes.

## Abbreviations

BMI: Body mass index
FFQ: Food frequency questionnaire
RCT: Randomised controlled trial
SD: Standard deviation
SMI: Severe mentally ill
TLM: Traffic light method for somatic screening and lifestyle
